# Genomic architecture of supergenes: connecting form and function

**DOI:** 10.1098/rstb.2021.0192

**Published:** 2022-08-01

**Authors:** Emma L. Berdan, Thomas Flatt, Genevieve M. Kozak, Katie E. Lotterhos, Ben Wielstra

**Affiliations:** ^1^ Institute of Biology Leiden, Leiden University, PO Box 9505, 2300 RA, Leiden, The Netherlands; ^2^ Naturalis Biodiversity Center, PO Box 9517, 2300 RA Leiden, The Netherlands; ^3^ Tjärnö Marine Laboratory, Department of Marine Sciences, University of Gothenburg, 45296 Strömstad, Sweden; ^4^ Department of Biology, University of Fribourg, Chemin du Musée 10, CH-1700 Fribourg, Switzerland; ^5^ Department of Biology, University of Massachusetts Dartmouth, 285 Old Westport Road, MA 02747, USA; ^6^ Department of Marine and Environmental Sciences, Northeastern University, 430 Nahant Road, Nahant, MA 01908, USA

**Keywords:** supergene, genomic architecture, structural variation

## Abstract

Supergenes are tightly linked sets of loci that are inherited together and control complex phenotypes. While classical supergenes—governing traits such as wing patterns in *Heliconius* butterflies or heterostyly in *Primula*—have been studied since the Modern Synthesis, we still understand very little about how they evolve and persist in nature. The genetic architecture of supergenes is a critical factor affecting their evolutionary fate, as it can change key parameters such as recombination rate and effective population size, potentially redirecting molecular evolution of the supergene in addition to the surrounding genomic region. To understand supergene evolution, we must link genomic architecture with evolutionary patterns and processes. This is now becoming possible with recent advances in sequencing technology and powerful forward computer simulations. The present theme issue brings together theoretical and empirical papers, as well as opinion and synthesis papers, which showcase the architectural diversity of supergenes and connect this to critical processes in supergene evolution, such as polymorphism maintenance and mutation accumulation. Here, we summarize those insights to highlight new ideas and methods that illuminate the path forward for the study of supergenes in nature.

This article is part of the theme issue ‘Genomic architecture of supergenes: causes and evolutionary consequences’.

## 1. The evolutionary puzzle of supergenes

A supergene is a set of tightly linked loci affecting a complex phenotype, with recombination between at least two different combinations of alleles (referred to as haplotypes hereafter) being reduced or absent [1–4]. As a consequence, these sets of alleles are inherited together, as if they were a single Mendelian locus (thus explaining the term ‘supergene’) [5]. Such an architecture can be beneficial when complex multi-trait phenotypes are under divergent selection in the presence of gene flow so that recombination opposes adaptation and speciation by homogenizing critical allele combinations [6–9]. The concept of a supergene is closely related to Dobzhansky's concept of ‘coadapted gene complexes', i.e. epistatic fitness interactions among loci maintained by reduced recombination, which he applied to chromosomal inversion polymorphisms [10–13]. The reduced recombination in the genomic region of the supergene splits the evolutionary trajectory of the region into at least two semi-independent branches [2,14] allowing for distinct multi-trait phenotypes (‘polymorphisms’: [15,16]) to segregate within a single population or to be easily transmitted across species. Although the potential evolutionary benefit of such a scenario was noted nearly a century ago [12,17], disentangling the complexities of supergene origin and evolution has remained a challenge.

New genomic, theoretical and bioinformatic methods now allow us to begin to understand the complex evolutionary history and diversity of supergenes [1,3,18,19]. In particular, the genomic revolution has highlighted a surprising amount of variation in the genomic architecture of supergenes. Genomic architecture plays a critical role in the continued evolution of supergenes as it dictates two of their key features: recombination rate and selective pressures on the supergene. First, large structural variants (SVs; e.g. chromosomal inversions, chromosomal fusions and indels), which are common supergene architectures, alter the effective recombination rate. However, different SVs vary in the extent and pattern of recombination suppression [20]. Second, selection can act on supergenes at three different scales, depending on genetic architecture: (i) there can be direct selection on the supergene itself, if potential breakpoints or deletions disrupt genes, create new genes [21] or separate loci from their regulatory elements; (ii) the allelic content of the haplotypes is also under selection, generating indirect selection at the level of the supergene through linkage disequilibrium; and (iii) there can be indirect selection on the supergene owing to the reduction in effective recombination between supergene haplotypes (i.e. selection because of a recombination load; [8,10,22,23]). Determining the relationship between genomic architecture and the evolution of supergenes is imperative for explaining and predicting supergene evolution and its consequences in natural populations [18].

The present collection of papers integrates (i) theoretical (including simulation) studies that generate predictions about supergene evolution, (ii) empirical studies that dissect genomic variation associated with supergenes, and (iii) synthetic opinion and review papers that tie together these diverse aspects of supergene evolution. Papers in this theme issue showcase the stunning architectural and taxonomic diversity of supergenes and cover a broad range of subjects relevant to our understanding of supergenes including local adaptation versus gene flow; parallel evolution; mutation accumulation and balanced lethality; sexually antagonistic selection; sex chromosome evolution; speciation; chromatin architecture and gene expression; and selfish genetic elements. In this introduction, we review the advances made by these papers, summarize the genomic architecture they uncover (§2) and detail how it connects to three major questions in supergene research: the role of supergenes in adaptation (§3), maintenance of supergene polymorphism (§4), and mutation accumulation in supergenes (§5). We highlight methodologies that can move the field forward and focus on outstanding questions in supergene evolution.

## 2. Genomic architecture of supergenes

Characterizing the genomic architecture of supergenes is the first step towards understanding their evolution. A major focal point is to comprehend the underlying genetic mechanisms of recombination suppression between supergene haplotypes. Certain genetic mechanisms can generate unbalanced gametes in supergene heterozygotes (e.g. chromosomal inversions or translocations), which has the two-pronged effect of generating underdominance as well as reducing effective recombination. For example, improper segregation of chromosomes in translocation heterozygotes can lead to the creation of aneuploid gametes (lacking critical genes) at a rate of 18–80% [24,25]. The extent of the reduction in effective recombination can also evolve over the lifetime of a supergene, through expansion or accumulation of additional SVs. Reconstructing the structural changes that generate recombination suppression is now possible using a combination of long-read sequencing and comparative genomics. Several papers in this issue dissect the genomic architecture of different supergenes, ranging from supergenes controlling colour polymorphism [21,26] to those contributing to female meiotic drive [27], in order to uncover the sources of recombination suppression.

Kim *et al*. [26] use trio-binning and long-read sequencing to construct chromosomal level assemblies of the BC supergene, associated with colour polymorphism in the African monarch butterfly, *Danaus chrysippus.* They find three alleles that differ dramatically in sequence, each containing multiple duplicated regions and inversions. Comparing BC supergene structure across the *Danaus* phylogeny, the authors reconstruct this supergene's evolutionary history and infer that it initially arose when a large genomic region was repeatedly duplicated approximately 7.5 Myr, with several inversions arising subsequently within this region of duplicated segments. Recombination suppression across the supergene probably spread through the fixation of these inversions, in addition to the genetic divergence of duplicated segments followed by differential loss of some of these duplicated regions. This second process of divergence and loss of duplicates may be a common contributor to supergene evolution.

Komata *et al.* [21] also use phylogenomic methods to understand the genomic architecture of supergenes. They focus on a supergene found in multiple species of *Papilio* butterflies that controls female-limited mimicry [28,29]. Two differentiated haplotypes of an autosomal genomic region (termed H and h) are associated with the mimetic and non-mimetic morphs, respectively. While the genomic architecture of H is known to include an inversion in *Papilio polytes,* no inversion is present in H in *Papilio memnon* [28,30,31]. Thus, this system is ideal for investigating the role of different genomic architectures in shaping supergene evolution, even when they lead to the same phenotype. Komata *et al.* [21] put together data from whole-genome resequencing studies, functional analyses and expression studies, to better understand the genomic architecture of the supergene in *P. memnon* and *P. polytes*. Using knock-down and expression studies, the authors are able to directly link genes within the supergene to different aspects of the female wing colour patterns. The authors show that both systems exhibit strong linkage disequilibrium in the supergene region as well as accumulation of repetitive sequences, a hallmark of low recombination regions [32–36]. Surprisingly, this indicates that the presence of an inversion may not have drastically changed the recombination rate in the supergene. Instead, the inversion in *P. polytes* seems to have generated a new gene, *U3X,* that regulates the expression of other loci within the supergene. Overall, this suggests that selection for the inversion may be acting on the breakpoints (i.e. direct selection on the inversion itself), as opposed to other characteristics such as reduced recombination.

Not all supergenes show suppressed recombination between haplotypes. Dufresnes *et al.* [37] investigate sex chromosomes that lack recombination suppression in the heterogametic sex. In most species, archetypal sex chromosomes come in two differentiated versions that do not recombine over a large stretch in the heterogametic sex (the male in XY systems such as mammals or the female in WZ systems such as birds). Because of this strong reduction in recombination, sex chromosomes are predicted to accumulate mutations at a faster rate than autosomes. This may cause sex chromosomes to behave like ‘supergenes of speciation’, with the non-recombining section accumulating genes responsible for reproductive isolation [38]. In fact, two rules of speciation—Haldane's rule (heterogametic hybrids show relatively higher sterility/inviability) and the large X-/Z-effect (X/Z chromosomes excessively accumulate incompatibilities relative to autosomes)—are based on the idea that sex chromosomes accumulate mutations. However, sex determination does not always follow the simple rules outlined above [39]. Amphibians lack the genomic architecture that suppresses recombination between the sex chromosomes. Dufresnes *et al*. [37] review this amphibian defiance of the classical laws of sex chromosome evolution and examine what it can teach us about the role of sex chromosomes as supergenes of speciation. The generally homomorphic sex chromosomes of amphibians do not appear to play a disproportionally large role in reproductive isolation as compared to the autosomes. These results are in contrast with support of both Haldane's rule and the large X-effect in insects, mammals, and fishes (who also have chromosomes without large-scale recombination suppression) [40]. Dufresnes *et al*. [37] highlight amphibians as a promising model to contrast with systems where sex chromosomes act as supergenes of speciation.

In addition to affecting linkage between loci, supergenes may also go beyond DNA changes to alter the epigenome, i.e. chromatin and methylation patterns. The ensuing consequences of supergenes on epigenomic modifications have been little explored up to now. In their paper, Wright & Schaeffer [41] explore the question of whether and how chromosomal rearrangements such as inversions might alter chromatin organization and modify gene expression; effects that might cause selection against these SVs. They map the breakpoints of seven pairs of inversion breakpoints in the fly *Drosophila pseudoobscura* to a map of topologically associated domains (TADs). TADs, which represent a form of higher order chromatin interactions, are self-interacting regions of the genome hypothesized to regulate gene expression [42–45]. Wright & Schaeffer [41] examine whether persisting inversion breakpoints are more likely to occur at boundaries between TADs and find that, indeed, breakpoints occur at TAD boundaries more often than expected by chance. Their study thus suggests that some inversion breakpoints might affect gene expression within TADs; this lends support to the hypothesis that position effects can contribute to the establishment of inversions.

Supergenes may also be selfish genetic elements, containing alleles that are specialized in over-representing themselves in the next generation through segregation distortion [46,47]. There are many well-known selfish supergenes, including *t*-haplotypes in mice [48], the *Sp* haplotype in the Alpine silver ant, *Formica selysi* [49] and *Segregation distorter* in *Drosophila melanogaster* [50]. Finseth *et al*. [27] provide evidence that the previously identified selfish centromere (D) in *Mimulus guttatus* is a supergene, segregating in multiple *Mimulus* populations in the Pacific northwest. They show that in several populations, D is a large (10–12 Mb) non-recombining region that leads to female meiotic drive and contributes to pollen inviability when homozygous (female-driving). D shows evidence of prior selective sweeps (possibly separate sweeps in two populations), but appears to be maintained within populations as a balanced polymorphism. Transcriptomes of individuals with and without the D allele indicate that many genes located within the supergene show reduction of expression in developing and reproductive tissues, suggesting many of the effects of D are *cis*-acting, similar to findings for other non-driving supergenes [51–53]. Finseth *et al*. [27] identify a number of potential linked candidate genes differing in expression that may lead to meiotic drive, including a centromere chaperone (NASP^SIM3^), further contributing to our knowledge of selfish supergenes.

## 3. Supergenes facilitate adaptation

Supergenes provide a mechanism that allows multiple favourable trait combinations to be maintained in the face of recombination. While the role of supergenes in adaptation has been well established [3–5,7,8,18,19,54], there remain fundamental questions about the ways in which supergenes facilitate adaptation. One of the most prominent of these questions is: do supergenes capture adaptive variants at the time of origin or gain these later? While some empirical studies find evidence that the accumulation of mutations inside a supergene over time caused it to establish and persist (‘gain’ or ‘accumulation’; [55]), others find that the supergene captured a beneficial combination of locally adapted alleles when it originated (‘capture’; [56,57]). Schaal *et al*. [58] use simulated data to explore the dynamics of capture and gain, while Stenløkk *et al*. [59] examine the evidence for capture versus gain using data from the Atlantic salmon.

Schaal *et al.* [58] address gaps in our understanding of capture and gain with whole-genome simulations of local adaptation with polygenic architectures and supergenes. Focusing on supergenes where recombination suppression is owing to inversions (i.e. inversion-based supergenes), their study relaxes assumptions made by the previous theory of fixed inversion size and location by allowing inversions of any size to mutate at any location in the genome. Importantly, this allows them to compare the characteristics of inversions involved in local adaptation (i.e. supergenes) to those that are not. The inversion-based supergenes that evolved in their simulations have many characteristics found in empirical studies (e.g. multiple old and large inversions that are outliers in genome scans, sometimes overlapping with other inversions), which highlights that empirical observations are consistent with a highly polygenic architecture and that supergenes do not necessarily need to contain any interesting large-effect genes to play an important role in local adaptation. Their simulations also show how, under polygenic architectures, supergenes can capture a small set of favourable alleles early in adaptation, which sets them on a trajectory to accumulate additional favourable alleles until an equilibrium level of local adaptation is achieved. Specifically, the simulations predict that adaptive inversion-based supergenes will harbour a significant proportion of the additive genetic variance in the trait under selection, but that a significant proportion will also be harboured in the collinear genome (i.e. the non-rearranged regions). Thus, these simulations produce specific hypotheses that can be tested by comparing the characteristics of supergenes to other inversions.

Stenløkk *et al*. [59] use empirical data to test for capture versus gain of adaptive alleles in Atlantic salmon. The authors take advantage of advances in sequencing and analysis to conduct a comparative analysis of all inversions segregating in the metapopulation of Atlantic salmon across the species range. For each inversion, they conduct an analysis of genes interrupted by the breakpoints, identified putatively deleterious mutations and identified single-nucleotide polymorphisms (SNPs) putatively under spatially heterogeneous selection by the environment. For this latter set of locally adapted SNPs, they were also able to determine if an allele was probably captured at the time of origin of the supergene or gained later, by comparing patterns to the ancestral state inferred from a closely related species. For the one inversion that shows clear patterns of local adaptation to the environment (e.g. a supergene), they find it is of a large size and both the capture and accumulation of adaptive variation was important in forming the supergene, findings that are consistent with the predictions of Schaal *et al.* [58].

A second critical question regarding the role of supergenes in adaptation is whether supergenes repeatedly arise. Observations of the same supergene are common in isolated populations of the same species or in closely related species and could result from several different evolutionary scenarios: multiple independent origins of the supergene, the presence of the supergene in a common ancestor or adaptive introgression of supergenes. Kay *et al.* [60] and Westram *et al*. [61] use both theoretical and empirical methods to differentiate between these possibilities.

Kay *et al.* [60] examine an elaborate ‘social polymorphism’ in the colony structure of ants found in many species. Ant colonies are either headed by a single queen (monogyne colonies) or by multiple queens (up into the hundreds; polygyne colonies). This social polymorphism, associated with a battery of phenotypic and life-history traits, reflects highly distinct evolutionary strategies, for which success depends on spatial environmental variation. Monogyne colonies fare better in habitat patches that have newly become available (and queens that found such colonies are, among many adaptations, better colonizers). Polygyne colonies have the upper hand in habitat patches that are already occupied (such colonies contain more workers and occur at higher densities). Remarkably, in the five ant lineages studied to date, it turns out that supergenes are involved in the social polymorphism, each with an independent origin. However, within some of these lineages, the social supergene has been co-opted by additional species within these lineages (in which the social polymorphism never evolved) via adaptive introgression [62]. Thus, both independent origins in addition to spread via adaptive introgression have been important in establishing the ant social supergenes.

The mechanisms behind the role of supergenes in parallel adaptation with gene flow is reviewed and modelled by Westram *et al*. [61]. The authors review multiple examples in which the same inversions are repeatedly implicated in local adaptation in different locations, indicating that supergenes may play a disproportionate role in parallel evolution. Using theory and simulation, they show how a supergene haplotype can function as an efficient ‘transport vehicle’ that can bring whole sets of adaptive alleles to a new location. Using models of local adaptation that span from a few patches with different environments to an environmental cline, they show how supergenes in one area of the species range can traverse space to facilitate parallel adaptation in another area of the species range. Their models predict that the presence of an inversion can create a supergene which speeds up the process of parallel adaptation and sometimes facilitates parallel adaptation when it would not have been possible otherwise. A limitation of the model is the assumption of only two adaptive loci. The authors highlight that an important direction for future research is to explore the dynamics with more polygenic architectures.

A third challenge in studying how supergenes facilitate adaptation is identifying adaptive variants in large regions of linkage disequilibrium generated by recombination suppression. Jay *et al*. [63] use a multivariate genome-wide association study (GWAS) to dissect the genetic basis of a supergene-based wing pattern polymorphism in the butterfly *Heliconius numata*. Within the supergene, which is itself composed of three chromosomal inversions, the authors identify several independent, putatively adaptive loci that are associated with different aspects of wing patterning. The results of Jay *et al.* [63] are consistent with a model whereby the inversions making up this supergene might have captured adaptive haplotypes, which predate the origin of the inversions. For some other loci, however, the results suggest that they might have been gained after the formation of the inversions (as modelled in Schaal *et al*. [58]).

Campoy *et al*. [64] review and analyse data from both GWAS and functional analyses to understand the phenotypic effects of two large inversions in humans that might likely represent supergenes. One of these inversions, 17q21.31, is associated with multiple complex phenotypes, including brain-related traits, red and white blood cells, lung function, male and female-specific traits, and disease risk. By combining data on nucleotide variation and gene expression, the authors pinpoint the role of three candidate genes (*CRHR1, KANLS1* and *MAPT*) that might underlie these phenotypes. The second inversion studied, 8p23.1, is also associated with several related phenotypes and gene expression differences; however, the complex breakpoint structure, and the apparent lack of genetic divergence within the inverted region away from the breakpoints, renders understanding the effects of this inversion challenging. Studying the properties of supergenes in humans is fundamentally important, not only for gaining evolutionary insights, but also for an improved understanding of how supergenes such as inversions impact human health and disease.

## 4. Maintaining supergene polymorphism

Many known supergenes are over 1 Myr old [18], begging the question of how these polymorphisms are maintained over large time scales in the face of drift and selection. Supergenes that persist over long time scales within a population are especially puzzling, given that supergene haplotypes often degrade by accumulating deleterious mutations [65–67]. Complex selective environments with multiple forms of balancing selection (e.g. overdominance, spatially variable selection, disassortative mating, antagonistic selection and negative frequency-dependent selection) are often necessary to protect supergene polymorphism. The non-trivial dynamics of polymorphism maintenance are examined theoretically by Tafreshi *et al.* [49] and Berdan *et al.* [68]. Dagilis *et al.* [69] take a mixed empirical and simulation approach, searching for signatures of sexually antagonistic selection, a particularly powerful form of balancing selection, in the three-spine stickleback.

Multiple forms of balancing selection are probably needed to protect polymorphism over long time scales, but we understand little about which combinations of selective pressures can achieve this. Tafreshi *et al*. [49] examine the evolutionary fate of a supergene given different combinations of balancing selection. They present a model based on an ancient supergene (approx. 20–40 Myr) in one particular ant species: the Alpine silver ant, *F. selysi*. This supergene has two haplotypes, *Sm* and *Sp,* that control colony structure in this haplodiploid species. In monogyne colonies, all queens and workers are *Sm/Sm* and males are *Sm*. In polygyne colonies queens and workers are *Sm/Sp* or *Sp*/*Sp*. However, the males produced in these colonies are only *Sp*, indicating the transmission ratio distortion caused by the *Sp* haplotype via a maternal killing effect [70]. Tafreshi *et al*. [49] find that this maternal killing effect creates a substantial challenge for polymorphism maintenance. In their model a stable polymorphism is only reached when there is both assortative mating, as well as large fitness differences between supergene genotypes. The narrow parameter space that allows polymorphism to be maintained highlights the complex dynamics necessary for supergenes to persist in nature.

One of the major challenges of supergene polymorphism maintenance is that the allelic content of the supergene haplotypes can shift over time. In particular, supergenes may accumulate deleterious mutations that lessen the selective edge of certain haplotype combinations [67]. Using simulations, Berdan *et al.* [68] examine the consequences of mutation accumulation on the maintenance of supergene polymorphism. Recessive deleterious mutations that are private to each supergene haplotype are masked in the heterozygote, generating associative overdominance (AOD; [71]). AOD describes a heterozygote advantage experienced by a neutral variant owing to selection on linked sites [71,72]. Berdan *et al*. [68] find that AOD aids invasion of a new supergene haplotype but that further mutation accumulation can destabilize the system, leading to the loss of the polymorphism. For this reason, the authors conclude that AOD alone is probably insufficient to maintain the polymorphism, and in most systems, multiple forms of balancing selection are probably necessary to maintain polymorphism over long time scales.

Another form of balancing selection that may maintain a supergene polymorphism within a population is sexually antagonistic selection, in which an allele that increases the fitness of males decreases the fitness of females, or vice versa. Dagilis *et al.* [69] investigate the possibility that sexually antagonistic selection is acting on stickleback sex chromosomes. Sexually antagonistic selection may lead to the spread of recombination suppression on sex chromosomes [73–75], but is often difficult to measure on older sex chromosomes where recombination suppression is already complete. Dagilis *et al.* [69] take advantage of the neo-Y chromosome that has been characterized in the Japan Sea stickleback (*Gasterosteus nipponicus*) [76], which formed through the fusion of the typical three-spine stickleback Y with an autosome. The authors compare sequences of male and female sticklebacks across the fused sex chromosome to determine if sexually antagonistic selection is driving genetic divergence in this region. Their results suggest that recombination suppression on the neo-Y chromosome has arisen relatively recently, perhaps only in the last few hundred thousand years. In the still recombining ‘pseudo-autosomal’ region of the neo-Y chromosome, the authors identify four genes involved in nervous system processes that show sequence divergence between males and females. However, using simulations, the authors found that demographic history alone could generate patterns of genetic divergence similar to these candidate regions. Thus, sexually antagonistic selection remains difficult to detect, even on recently evolved and recombining sex chromosomes.

## 5. Mutation accumulation in supergenes

The reduction in effective recombination between supergene haplotypes can be viewed as a double-edged sword: it can facilitate adaptive processes when beneficial alleles are brought together, but might also speed up the accumulation of deleterious mutations [18]. This is because suppressed recombination also reduces the efficacy of purifying selection. The interplay between recombination, drift and linked selection has long-term consequences for the fate of supergenes, which are only now being fully recognized. Advances in the computational power of forward simulations (e.g. SLiM, [77]) are enabling us to theoretically model and predict the evolutionary consequences of supergenes in unprecedented detail. Berdan *et al.* [68] use simulations to look at mutation accumulation in supergenes and the ensuing consequences, while Stenløkk *et al*. [59] look for signatures of this mutation accumulation in Atlantic salmon.

Berdan *et al.* use simulations in SLiM [77,78] to examine the costs and consequences of mutation accumulation in supergenes. While AOD owing to fixed recessive deleterious alleles could facilitate the invasion of a new supergene haplotype and the origin of the supergene polymorphism, continued mutation accumulation strongly threatened the polymorphism. Asymmetry in mutational load between the two haplotypes often leads to the least fit haplotype degenerating more rapidly, which makes the polymorphism sensitive to loss through drift. This is because a high mutational load drives down the frequency of the haplotype, which translates into purifying selection being far less effective and an increase in mutation accumulation. This further decreases the frequency of the more mutationally loaded haplotype, which further decreases the efficacy of purifying selection, in a feedback loop [65]. The authors found that even small differences in load between the two haplotypes could trigger this feedback loop, leading to the loss of the polymorphism. The feedback loop is driven by strong differences in the efficacy of purifying selection in the two haplotypes, which is tied to differences in effective population size. The only way for both haplotypes to degrade at a similar rate is a strong reduction in population size so that drift becomes the main evolutionary force. In small populations, purifying selection will be less effective in general and in the fitter haplotype in particular. This decreases the differential in the efficacy of purifying selection between haplotypes. When both haplotypes degrade at the same rate, they can reach an evolutionary stable state known as a balanced lethal system. As both supergene homozygotes are inviable, and only heterozygotes contribute to subsequent generations, the polymorphism can never be lost without the extinction of the population. This maladaptive situation has been described in plants, insects and vertebrates [79,80].

Stenløkk *et al*. [59] directly test for mutation accumulation in inversions segregating in the metapopulation of Atlantic salmon. For each inversion they assess accumulation of deleterious mutations (or lack thereof), and analyse change through time, by comparing the focal inversion to its ancestral state of inversions in a sister species. They find a lack of evidence for the accumulation of deleterious mutations as predicted by theory, but conclude that this is probably owing to the young age of the inversions.

## 6. Concluding remarks

By bringing together diverse empirical and theoretical studies of supergenes, this theme issue provides new insights into supergene evolution. While supergenes are a classical subject in evolutionary biology, investigating the evolution of supergenes has only really taken off within the twenty-first century [3,18,19,54]. This renewed interest in supergenes, driven by new methodological and theoretical developments, has revealed that supergenes abound in nature and underlie many fantastically complex phenotypes. It is clear that the evolution of supergenes is incredibly complicated, as both the external environment as well as the allelic content of different supergene haplotypes shift over time. Multiple different processes and selective pressures must then work together to maintain these polymorphisms over long time scales. However, the emergence and subsequent interaction of these different selective pressures over the evolutionary history of a supergene remain unknown. As the papers in this theme issue show, integration of both theoretical and empirical techniques can help bring these puzzles into focus. By bringing together work on diverse approaches, species and supergene structures, we hope this theme issue stimulates future discussion and research on the topic of supergene evolution.

## Data Availability

This study has no associated data.
